# Use of Rideshare Services to Increase Participant Recruitment and Retention in Research: Participant Perspectives

**DOI:** 10.2196/11166

**Published:** 2019-04-08

**Authors:** Eleanor Ladd Schneider Leavens, Elise Marie Stevens, Emma Irene Brett, Neil Molina, Thad Ryan Leffingwell, Theodore Lee Wagener

**Affiliations:** 1 Oklahoma Tobacco Research Center Oklahoma City, OK United States; 2 Oklahoma State University Department of Psychology Stillwater, OK United States; 3 University of Oklahoma Health Sciences Center Department of Pediatrics Oklahoma City, OK United States

**Keywords:** rideshare service, recruitment, retention, attrition, transportation

## Abstract

**Background:**

Recruitment and retention of participants are important factors in empirical studies. Methods that increase recruitment and retention can reduce costs and burden on researchers related to the need for over-recruitment because of attrition. Rideshare services such as Uber and Lyft are a potential means for decreasing this burden.

**Objective:**

This study aimed to understand the role rideshare utilization plays in participant recruitment and retention in research trials.

**Methods:**

Data are presented for a study (*N*=42) in which rideshare services were utilized for participant transportation to and from study visits during a 2-session, in-laboratory research study.

**Results:**

Retention at visit 2 was greater than 95% (42/44) in the initial study. In a follow-up survey of the participants from the original trial, participants (N=32) reported that the rideshare service was an important reason they returned for all study visits. Participants reported whether they would prefer differing levels of additional monetary compensation or a ride from a rideshare service. When the additional compensation was less than US $15, participants reported a preference for the rideshare service.

**Conclusions:**

Rideshare services may represent a relatively low cost means for increasing study retention. Specifically, findings indicate that rideshare services may not be crucial for initial participant recruitment but for their retention in multi-visit studies.

## Introduction

### Background

In health, behavioral, and social sciences, human laboratory and randomized controlled trials are imperative to further science and interventions [1-4]. Two critical aspects of research studies are recruitment and retention [5]. Recruitment is the process by which potential research participants are made aware of and then enrolled in the study [5], whereas retention refers to participants staying in the study and completing study visits [6]. When researchers fail to recruit and retain participants, findings can be invalid, inconclusive, and insufficient to answer research questions [7-9]. In addition, attrition can be costly and result in using greater resources, extending studies, and in some cases, terminating studies prematurely [10].

Research suggests that telephone reminders and financial incentives are advantageous ways to recruit and retain participants [11]. Specifically, studies have shown that participants increase their willingness to participate when compensation increases [12,13], regardless of the risk of adverse events that may result from study participation [12]. Recruitment and retention strategies that reduce participant burden, including costs associated with transportation, are likely to impact desirability of study participation.

It is also likely that the content area impacts recruitment and retention of participants. On one hand, because of the unique challenges associated with retaining participants in substance abuse studies, the threshold for good retention in these studies has been set relatively low (70%) [14]. However, in studies utilizing alcohol and tobacco administration, retention rates are relatively high (90%-96%) [15,16]. However, these referenced studies also required participants to complete baseline screening visits and did not include participants who did not return following screening, which likely increased retention rates.

A recent study proposed a conceptual framework specific to improving recruitment and retention in tobacco and alcohol research, including (1) creating a team mindset that promotes regular and positive communication with participants, (2) leveraging technology, and (3) increasing efforts to contact nonresponsive participants [17]. Applying such techniques as applicable to a specific sample may improve retention by establishing an alliance between the study team and the participant and reducing participant burden. In addition, literature on retention suggests that using more strategies across several separate categories (eg, visit characteristics, study personnel, and nonfinancial incentives) will result in improved retention [18].

With the advent, popularity, and low cost of rideshare services such as Uber and Lyft, it is important to understand if and how such services may represent a novel and advantageous strategy to recruit and retain participants. Past studies among low-income urban participants have shown that reimbursing for taxi services was not always an effective method for recruitment and retention because of unreliability of taxis [8]. However, rideshare services may be more advantageous given the researcher’s capability to order the rides remotely at the scheduled time, track participants’ rides to the study location, and set up an account to facilitate hassle-free payment for the service. Use of rideshare services may also increase safety for studies in which acute intoxication is necessary and driving would put participants in harm’s way. Finally, use of rideshare services reduces participants’ burden and is consistent with the suggestions within Smith and colleagues’ framework for increasing retention [17].

### Objectives

The aim of this study was to assess participants’ perceptions of the use of a rideshare service in terms of the impact it had on decisions to return to study visits in a recently completed, multi-visit study. We also examined how providing rideshare services in future studies would influence participants’ decisions to participate. Finally, we aimed to understand whether differing levels of additional compensation or rideshare services would be better for recruitment and retention.

## Methods

### Participants and Procedure

This study recruited participants from a recently completed study [19]. The purpose of the original study was to understand the impact of acute alcohol intoxication on waterpipe smoking patterns and toxicant exposure. The completed research study recruited 21 dyads (*N*=42) of current waterpipe smokers and drinkers for a 2-session, in-laboratory study. Each visit included survey completion, 2 blood draws, breath tests (breath alcohol concentration and carbon monoxide), and alcohol or placebo beverage administration, followed by a waterpipe smoking session lasting up to 2 hours. Of the 44 participants, 42 (95.5%) were retained in the study. Retention methods included regular calls to participants, relationship building between research staff and participants, fair compensation (US $125 per visit) with a bonus (US $20) for completing both study visits, and transportation to and from study visits via a rideshare service.

Participants who completed both study visits in the original study were invited to provide feedback on their experiences, with the primary aim of understanding the role of the provision of a rideshare service in their choice to complete both study visits. Before completing study procedures, participants provided informed consent. All data were collected remotely via a brief, Web-based survey. Participants were compensated with a US $5 gift card. Of the 42 participants who completed the original study, 32 (mean age 25.7, SD 3.0; 58% male; 79% white) completed this study. The university’s institutional review board approved all study procedures.

### Use of Rideshare

All participants were required to utilize rideshare services (eg, Uber and Lyft) for their transportation to and from the research site. This requirement not only facilitated the double-blind nature of the study but also enhanced participant safety following alcohol administration. An Uber account was created for the purpose of this research, and all rides were placed from this account. Participants were required to be picked up from and dropped off at their home address to limit the likelihood participants would drive following participation. Pick up from work or other locations was not allowed because of the likelihood that participants would be dropped off at their car following study participation. Ten to 20 min before the scheduled study visit, the research staff contacted participants via phone to ensure they were ready for the ride request to be placed. If confirmed, research staff placed a request for a rideshare service to pick up the participant and bring them to the laboratory. As participants confirmed they were available for pick up, only 2 rides necessitated cancelling during the study. One cancellation was due to the participant having difficulty locating the vehicle, and the other was due to the participant necessitating additional time. Both rides were able to be rescheduled soon after the original ride was declined. Following each visit, research staff placed a request for the rideshare service to pick up the participants at the laboratory and take them to their home addresses.

**Table 1:** Importance of recruitment and retention strategies compared with provision of rideshare services (N*=* 32).

Variables		Recruitment and retention strategies		
		Mean (SD)	*t* test	*P* value
**Reasons for study completion**				
	Rideshare service was provided^a^	5.75 (1.70)	—^b^	—
	The study visits were in the evening	5.47 (1.90)	0.64	.53
	The staff was nice	6.47 (1.30)	−2.26	.03
	I received reminder short message service (SMS) text messages	4.97 (1.84)	2.60	.01
	I received reminder calls from staff	4.66 (1.81)	2.86	.01
	Alcohol was provided at study visits	4.56 (2.23)	2.65	.01
	Hookah was provided at study visits	5.28 (1.42)	1.17	.25
	The compensation was fair	6.28 (0.96)	−1.78	.08
	I would feel bad if I did not attend all visits	5.72 (1.69)	0.07	.94
	I got to complete study with my friend	6.31 (0.90)	−1.74	.09
	The study visits were fun	6.47 (0.88)	−2.35	.03
**Intentions for future study participation**				
	Rideshare service was provided^a^	4.13 (0.75)	—	—
	The study visits were in the evening	4.38 (0.71)	−1.61	.12
	The staff was nice	4.56 (0.62)	−2.95	.01
	I received reminder SMS text messages	4.06 (0.67)	0.44	.66
	I received reminder calls from staff	3.88 (0.79)	1.54	.13
	Alcohol was provided at study visits	4.25 (0.84)	−0.89	.38
	Hookah was provided at study visits	4.13 (0.79)	0.00	>.99
	The compensation was fair	4.81 (0.40)	−4.98	<.01
	I got to complete study with my friend	4.63 (0.55)	−3.22	.003
	The study visits were fun	4.69 (0.54)	−3.97	<.001

^a^Reasons for study completion were measured on a 1 (*not at all important*) to 7 (*extremely important*) scale. Intention for future study participation were measured on a 1 (*strongly disagree*) to 5 (*strongly agree*) scale.

^b^All *t* tests compared use or rideshare to each other strategy, therefore no values are included for the rideshare test as there is no comparator.

#### Confidentiality Considerations

Participants consented to the use of the rideshare service at screening and via the main consent form. Specifically, participants were informed a rideshare service would be utilized for transportation but that only their first name and address would be provided to the rideshare service. Drivers were not informed that participants were enrolled in a research study. Participants were further informed that the researcher would place the ride requests but that their participation remained voluntary and could be discontinued at any time and with no penalty to participants.

### Measures

#### Reasons for Study Completion

Participants completed 11 items assessing the importance of different recruitment and retention strategies in their decision to complete both visits of the original study. Items were rated on a Likert scale from 1 (*not at all important*) to 7 (*extremely important*). See Table 1 for a complete list of recruitment and retention strategies that were assessed.

#### Intention for Future Study Participation

Intention for future study participation was measured by 10 items. Participants reported their agreement with each item. Each item completed the sentence beginning with “I would participate in another study like this if...” Response options ranged from 1 (*strongly disagree*) to 5 (*strongly agree*). See Table 1 for a complete list of recruitment and retention strategies that were assessed.

#### Multiple-Choice Procedure

To further understand participant preferences for rideshare services in research, participants completed a multiple-choice procedure (MCP) [20] task in which they were presented with the choice between varying levels of additional compensation or a ride to and from the study via a rideshare service. The purpose of this item was to assess participants’ interest in the rideshare service versus additional compensation in some future study. The prompt did not specify the amount of compensation for the hypothetical future study, only the additional compensation amounts versus rideshare provision. Participants were instructed: “ *Imagine you have been invited to participate in an in-person research study similar to the one you previously completed in our laboratory. Below is a list of monetary values and free Uber rides. Please choose between the monetary value and Uber ride for each set. In other words, for each set, would you rather have the money or a free Uber ride to your study visit?”* Monetary values ranged from US $0.00/free to US $1000. The crossover value, or point where a switch in preference occurred from the rideshare service to the monetary value, was used to indicate the importance of rideshare compared with additional compensation.

### Data Analysis

For outcomes including reasons for returning to study visits and intentions for future study participants, means and SDs were calculated for each item. The mean for the rideshare item was compared with the means for all other recruitment and retention strategy items using paired samples *t* tests. Significance was set to *P*<.05. For the MCP, descriptive information regarding the frequency of preferences for additional compensation or the rideshare service is presented.

## Results

### Reasons for Study Completion

Participants reported that the provision of transportation via a rideshare service was an important reason they returned for all study visits (mean 5.75, SD 1.70). Participants reported that the provision of a rideshare service was more important in their decision to complete all visits than reminder short message service (SMS) text messages from staff (mean 4.97, SD 1.84; *t*_31_=2.60; *P*=.01), reminder calls from staff (mean 4.66, SD 1.81; *t*_31_=2.86; *P*=.007), and alcohol being provided at study visits (mean 4.56, SD 2.23; *t*_31_=2.65; *P*=.01). However, compared with the provision of a rideshare service, participants rated the staff being nice (mean 6.47, SD 1.30; *t*_31_=−2.26; *P*=.03) and the visits being fun (mean 6.47, SD 0.88; *t*_31_=−2.35; *P*=.03) as more important in their completion of all study visits.

### Intention for Future Study Participation

Overall, participants reported that they would participate in a similar study that offered rideshare services in the future (mean 4.13, SD 0.75). However, compared with other recruitment strategies, participants reported a preference for nice staff (mean 4.56, SD 0.62; *t*_31_=−2.95; *P*=.006), fair compensation (mean 4.81, SD 0.40; *t*_31_=4.98; *P* ≤.001), the option to participate with a friend (mean 4.63, SD 0.55; *t*_31_=3.22; *P*=.003), and fun study visits (mean 4.69, SD 0.54; *t*_31_=−3.97; *P* ≤.001) compared with the provision of rideshare services to and from study visits. See Table 1 for complete results.

### Multiple-Choice Procedure

**Figure 1:**
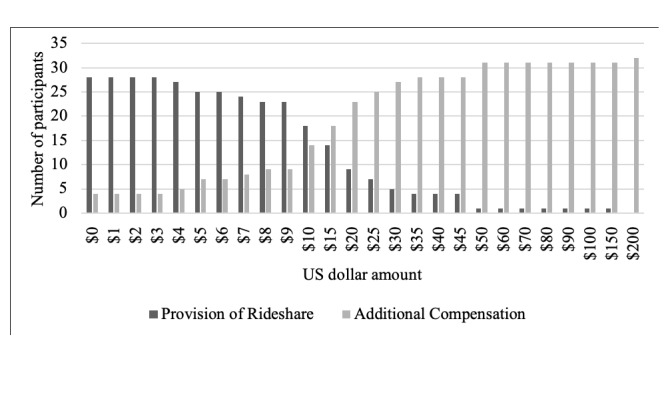
Multiple Choice Procedure—Rideshare versus compensation crossover.

The crossover point on the MCP was observed from US $10 to US $15 such that at levels of additional compensation below US $15, participants showed a preference for the rideshare service. However, participants showed a preference for compensation when the monetary value exceeded US $15 (see Figure 1).

## Discussion

### Principal Findings

This study is the first to examine participants’ perceptions of the use of a rideshare service on their decision to return to study visits and participate in future studies. Recruitment and retention are 2 highly important aspects of successful research [5], and rideshare services may represent a new way to increase participant engagement. In this study, provision of rideshare services was reported to be an important reason participants completed all visits of the original study. Participants also reported they would be interested in completing a future study that provided transportation via a rideshare service. Provision of rideshare services was rated as more important for continued participation than other common recruitment and retention strategies, such as providing reminders for study visits. Alternatively, when considering strategies that would be important in their decision to participate in future studies, participants rated fair compensation and an enjoyable study visit as more important than provision of rideshare services. These seemingly discrepant findings may indicate that rideshare services may not be crucial for the initial recruitment of participants into studies, but for their retention in studies that require more than 1 on-site visit.

Past research has shown that provision of taxi services is not an effective method for recruitment or retention because of the unreliability of taxis [8], but participants in this study reported provision of rideshare was an important reason they returned for study visits, indicating rideshare may be preferred. This difference may be because of reduced participant burden because the researcher is facilitating provision of the ride. In addition, as app-based rideshare services continue to increase in popularity, more people will have experience using them, and thus, be more comfortable with their utilization in research studies. It is currently unknown how perceptions of rideshare versus taxi services may or may not account for these discrepant findings. The use of rideshare services may be a cost-effective way to retain participants, particularly given the high costs both to the researcher and the integrity of the study associated with attrition [7-9]. We investigated the trade-off between providing additional compensation and providing transportation via a rideshare service. The crossover point may indicate that additional compensation is more beneficial than provision of rideshare services at values greater than US $15 but that rideshare services may be more effective if participants live close to the study site and rides cost less than US $10 per participant.

The results of this study, coupled with the outstanding retention rate (95%; 42/44) in the original study, suggest that provision of transportation via rideshare services may be a means for increasing retention that should be shared with other research teams. The potential decreased costs and burdens on research staff related to a decreased need to over-recruit to address attrition may result in significant saved costs. Specifically, saved costs would include those associated with recruiting and compensating additional participants because of decreased attrition. Furthermore, compared with taxis, rideshare services are often less expensive, resulting in additional saved costs. Research staff can also be aware of exact arrival time of participants, given that the rideshare services provide real-time locations of the transportation. In addition, it is likely that the avoided costs of over-recruiting to replace participants lost to follow-up outweigh the costs associated with providing rideshare services. This strategy may be particularly helpful for recruiting and retaining individuals with inconsistent methods of transportation or financial barriers that would make obtaining reliable transportation and attending study visits difficult. Furthermore, utilization of rideshare services in addiction studies in which substance administration is required can reduce additional time and resource burden on researchers. Rideshare services represent a means by which to ensure participants arrive home safely. In the case of alcohol administration studies, particularly those using low alcohol doses, use of rideshare services may limit the need for research staff to remain in the lab with participants until their breath alcohol concentration is 0.000.

### Limitations

Although this study is an important step in understanding the integration and use of rideshare services in research, this study has 2 primary limitations. First, we did not utilize a control and are therefore unable to compare differences in recruitment and retention in studies that did and did not use rideshare services. However, the study demonstrated exceptional retention relative to typical studies in the literature, indicating the retention procedures were successful. It is impossible to conclude with strong inference that rideshare was the critical ingredient, but participant reports are consistent with this conclusion. Second, this study may not generalize to other study designs, studies with different aims and methods, or more highly diverse participant populations. Third, although approximately 76% (32/42) of eligible participants participated in this study, we were not able to make conclusions regarding those who chose not to participate in this study and similarly did not assess reasons for discontinuing participation in the original trial.

### Conclusions

Despite the large number of studies that require multiple in-lab visits, there has been little research on novel retention and recruitment strategies, an area that is critical for the success of such research. As illustrated in this study, use of rideshare services for in-lab studies may be a worthwhile strategy for increasing retention in research. Utilization of rideshare services should be considered to supplement existing and established methods for improving study recruitment and retention in multi-visit studies.

## Abbreviations

MCP: multiple-choice procedure
SMS: short message service
